# Glycosylation of the Hemagglutinin Protein of H5N1 Influenza Virus Increases Its Virulence in Mice by Exacerbating the Host Immune Response

**DOI:** 10.1128/JVI.02215-16

**Published:** 2017-03-13

**Authors:** Dongming Zhao, Libin Liang, Shuai Wang, Tomomi Nakao, Yanbing Li, Liling Liu, Yuntao Guan, Satoshi Fukuyama, Zhigao Bu, Yoshihiro Kawaoka, Hualan Chen

**Affiliations:** a State Key Laboratory of Veterinary Biotechnology, Harbin Veterinary Research Institute, Chinese Academy of Agricultural Sciences, Harbin, People's Republic of China; b Division of Virology, Department of Microbiology and Immunology, Institute of Medical Science, University of Tokyo, Tokyo, Japan; c Department of Pathobiological Sciences, School of Veterinary Medicine, University of Wisconsin-Madison, Madison, Wisconsin, USA; d Department of Special Pathogens, International Research Center for Infectious Diseases, Institute of Medical Science, University of Tokyo, Tokyo, Japan; Wake Forest University

**Keywords:** H5N1 viruses, glycosylation, mouse, pathogenicity

## Abstract

The highly pathogenic avian influenza (HPAI) H5N1 viruses continue to circulate in nature and threaten public health. Although several viral determinants and host factors that influence the virulence of HPAI H5N1 viruses in mammals have been identified, the detailed molecular mechanism remains poorly defined and requires further clarification. In our previous studies, we characterized two naturally isolated HPAI H5N1 viruses that had similar viral genomes but differed substantially in their lethality in mice. In this study, we explored the molecular determinants and potential mechanism for this difference in virulence. By using reverse genetics, we found that a single amino acid at position 158 of the hemagglutinin (HA) protein substantially affected the systemic replication and pathogenicity of these H5N1 influenza viruses in mice. We further found that the G158N mutation introduced an N-linked glycosylation at positions 158 to 160 of the HA protein and that this N-linked glycosylation enhanced viral productivity in infected mammalian cells and induced stronger host immune and inflammatory responses to viral infection. These findings further our understanding of the determinants of pathogenicity of H5N1 viruses in mammals.

**IMPORTANCE** Highly pathogenic avian influenza (HPAI) H5N1 viruses continue to evolve in nature and threaten human health. Key mutations in the virus hemagglutinin (HA) protein or reassortment with other pandemic viruses endow HPAI H5N1 viruses with the potential for aerosol transmissibility in mammals. A thorough understanding of the pathogenic mechanisms of these viruses will help us to develop more effective control strategies; however, such mechanisms and virulent determinants for H5N1 influenza viruses have not been fully elucidated. In this study, we identified glycosylation at positions 158 to 160 of the HA protein of two naturally occurring H5N1 viruses as an important virulence determinant. This glycosylation event enhanced viral productivity, exacerbated the host response, and thereby contributed to the high pathogenicity of H5N1 virus in mice.

## INTRODUCTION

The highly pathogenic avian influenza (HPAI) H5N1 viruses continue to circulate in nature and threaten animal and human health. HPAI H5N1 viruses have been found in the domestic poultry or wild birds of more than 60 countries across three continents (1) and have become enzootic in poultry. Their effects have taking a huge toll on the poultry industry in many countries, including Bangladesh, China, Egypt, India, Indonesia, and Vietnam (2). Since the first recognized human cases of HPAI H5N1 virus infection in Hong Kong in 1997, the World Health Organization has reported 854 confirmed human infection cases as of July 2016, with a fatality rate of over 50%. HPAI H5N1 viruses were reported to have limited potential for human-to-human transmission (3–5). Recent studies, however, have demonstrated that HPAI H5N1 viruses can be transmitted in ferrets or guinea pigs by respiratory droplets if they acquire certain mutations or reassort with pandemic 2009 H1N1 virus (6–8). Accordingly, it is essential for us to gain a thorough understanding of the pathogenic mechanisms of these viruses in order to develop more effective control strategies.

Genetic manipulation of influenza viruses and studies with different mammal models have led to the identification of several viral markers that are associated with virulence and pathogenesis (9–16). Most notably, the reconstruction of the 1918 Spanish pandemic influenza virus and studies of HPAI H5N1 viruses have clearly established the complexity and multigenic characteristics of influenza virus pathogenesis. Hemagglutinin (HA), neuraminidase (NA), and the viral RNA polymerase complex play important roles in the high virulence of the 1918 pandemic influenza virus in mice (17, 18). The cleavage site of HA plays crucial roles in the systemic replication and lethal infection of the H5 and H7 subtypes of influenza viruses in chickens (19) and mammals (10, 20). Mutations in the M1 protein also contribute to the virulence of H5N1 influenza viruses in mice (9). Certain amino acids or regions of the NS1 protein subvert the antiviral immune response of the host and are essential to the pathogenicity of H5N1 influenza viruses in mice (11, 15, 21). The amino acids at positions 627 and 701 of PB2 are principal determinants of the high virulence of H5N1 influenza viruses in mammals (10, 12); 30% and 5% of human H5N1 isolates have been reported to possess 627K and 701N in their PB2 proteins, respectively (http://www.ncbi.nlm.nih.gov/). Moreover, the amino acid at position 591 of PB2 is reported to be important for the efficient replication of pandemic H1N1 viruses in humans (22) and to substantially increase the pathogenicity of an avian H5N1 virus in mice (23). The PA protein directly contributes to the virulence of H5N1 avian influenza viruses in domestic ducks (24) and mice (25, 26). Thus, although we are acquiring important data in this area, the mechanism of host range and the virulent determinants of H5N1 influenza viruses have yet to be fully explored.

Disease outcomes reflect the battle between pathogens and hosts. In addition to viral intrinsic factors, host-specific traits and differences in host immune responses can ameliorate or exacerbate both the infection and prognosis. Likewise, host responses contribute to the pathogenesis of H5N1 viruses in mammals. Many studies have demonstrated that dysregulation of the innate immune responses during H5N1 influenza virus infection increase disease severity (27–30).

Previously, we characterized the genetic and biological diversity of HPAI H5N1 viruses in Vietnam (31), and found that two viruses, A/chicken/Vietnam-Bac Lieu/1214/2007 (CK/1214) and A/chicken/Vietnam-Ca Mau/1180/2006 (CK/1180), shared similar genetic backgrounds, belonging to the same genotype, but displayed substantially different levels of virulence in mice (31, 32). In the present study, we used reverse genetics to identify the molecular determinants for the different virulence of these two viruses in mice and explored the possible underlying mechanisms.

## RESULTS

### Rescued H5N1 viruses retain the biological properties of wild-type viruses.

**FIG 1:**
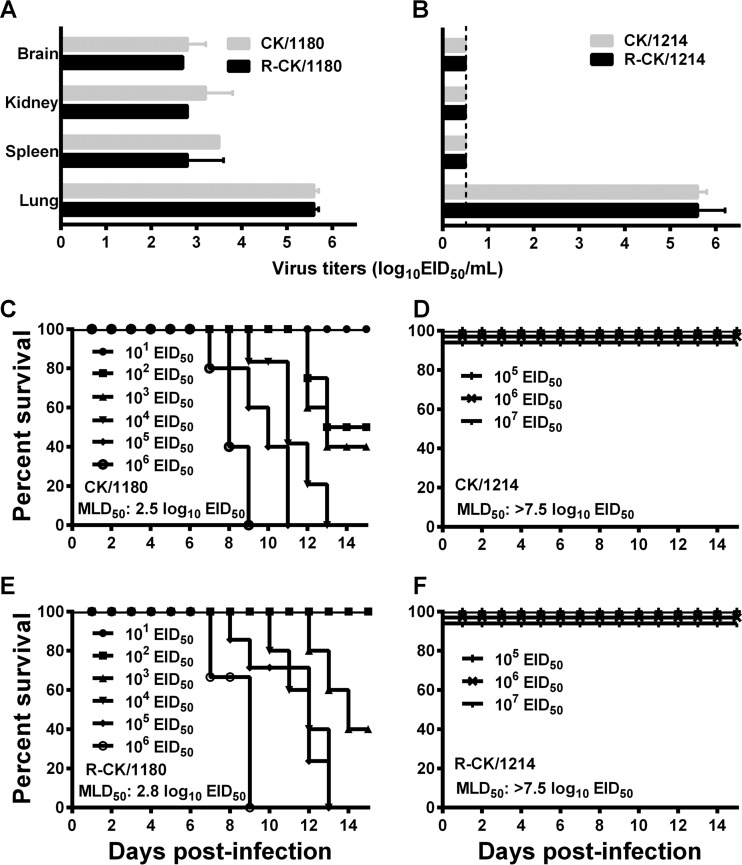
Replication and lethality of the CK/1180 and CK/1214 viruses in mice. (A and B) Six-week-old SPF BALB/c mice (three/group) were inoculated intranasally with 10^6^ EID_50_ of each virus, and organs were collected on day 3 postinfection for virus titration in eggs. Data are means ± standard deviations (SD). (C to F) Mortality assessment of mice infected with different H5N1 viruses: CK/1180 (C), CK/1214 (D), R-CK/1180 (E), and R-CK/1214 (F).

In our previous studies (31, 32), we characterized the pathogenicity and viral replication of CK/1180 and CK/1214 in mice and found that CK/1180 systemically replicated and showed high pathogenicity in mice, with a 50% mouse lethal dose (MLD_50_) of 2.5 log_10_ 50% egg infectious doses (EID_50_), but CK/1214 replicated only in lung tissue and displayed low virulence in mice. In this study, we found that even 10^7^ EID_50_ of CK/1214 did not kill mice (MLD_50_ > 7.5 log_10_ EID_50_) (Fig. 1). By using constructed plasmids, we rescued the CK/1180 and CK/1214 viruses, designated R-CK/1180 and R-CK/1214, respectively. After confirming the sequences of the rescued viruses, we tested their replication and lethality in mice. Similar to the wild-type virus, the rescued R-CK/1180 replicated systemically (Fig. 1A and Table 1) and was highly pathogenic in mice (Fig. 1C and E). R-CK/1214 virus, like its wild-type counterpart, did not kill mice and replicated only in the lungs of the organs tested (Fig. 1B, D, and F). These results indicate that the rescued viruses maintained the biological properties of the wild-type viruses. These two viruses share the same NP gene product at the amino acid level and differ by only 24 amino acids in their PB2, PB1, PA, PA-X, HA, NA, M1, M2, NS1, and NS2 proteins (Table 2).

**TABLE 1:** Replication of wild-type and rescued viruses in BALB/c mice inoculated intranasally with 10^6^ EID_50_ of virus (in 50 μl)^*a*^

Virus	Virus titer (log_10_ EID_50_/ml), mean ± SD					MLD_50_ (log_10_ EID_50_)	Fold change in attenuated virulence^*b*^
	Lung	Spleen	Kidney	Brain	NT		
CK/1214	5.6 ± 0.2	−	−	−	ND	>7.5	No
R-CK/1214	5.6 ± 0.6	−	−	−	2.9 ± 0.3	>7.5	No
CK/1180	5.6 ± 0.1	3.5 ± 0.0	3.2 ± 0.6	2.8 ± 0.4	ND	2.5	No
R-CK/1180	5.6 ± 0.1	2.8 ± 0.8	2.8 ± 0.0	2.7 ± 0.0	3.8 ± 0.7	2.8	No
CK/1180-1214PB2	5.6 ± 0.1	+	1.3 ± 0.1	+	ND	4.5	50
CK/1180-1214PB1	5.7 ± 0.3	3.5 ± 0.2	2.8 ± 0.1	2.5 ± 0.2	ND	3.5	5
CK/1180-1214PA	5.6 ± 0.1	3.5 ± 0.0	2.3 ± 0.2	2.5 ± 0.0	ND	3.6	6
CK/1180-1214HA	6.4 ± 0.2	+	−	−	3.4 ± 0.1	6.4	3,981
CK/1180-1214NA	5.8 ± 0.1	1.9 ± 0.4	1.5 ± 0.0	+	ND	3.6	6
CK/1180-1214 M	6.3 ± 0.2	2.2 ± 1.5	1.2 ± 0.4	0.8 ± 0.0	ND	3.5	5
CK/1180-1214NS	6.4 ± 0.2	2.6 ± 0.4	1.8 ± 0.4	−	ND	4.2	25
DK/49	6.7 ± 0.5	4.4 ± 0.1	4.2 ± 0.6	3.4 ± 0.3	4.8 ± 0.3	0.5	No
DK/49-HA-N158G	6.1 ± 0.3	3.9 ± 0.5	2.9 ± 0.5	2.0 ± 0.6	3.9 ± 0.3	0.8	2

a Six-week-old BALB/c mice were inoculated intranasally with 10^6^ EID_50_ of each virus. Three mice from each group were killed on day 3 postinfection, and virus titers were determined in samples of lung, spleen, kidney, and brain in eggs. −, no virus was isolated from the sample; +, virus was isolated from undiluted sample. NT, nasal tissue; ND, no detection.

b Fold change in attenuated virulence of single-gene reassortants in mice compared with that of R-CK/1180. No, no comparison with R-CK/1180.

**TABLE 2:** Amino acid differences between avian influenza viruses CK/1214 and CK/1180

Viral protein	Amino acid position	Amino acid in:	
		CK/1214	CK/1180
PB2	251	Arg	Lys
	463	Val	Ile
	647	Thr	Ile
PB1	152	Ser	Thr
	323	Met	Thr
	618	Val	Glu
PA	5	Val	Ala
	228	Asn	Asp
	357	Lys	Thr
	445	Tyr	His
	611	Phe	Leu
	673	Arg	Lys
PA-X	5	Val	Ala
	224	Glu	Ala
HA (H3 numbering)	158	Gly	Asn
NA	250	Asn	Asp
	253	Asp	Asn
M1	168	Ile	Thr
M2	16	Glu	Glu
	65	Thr	Arg
	68	Ile	Val
NS1	73	Tyr	Ser
	204	Val	Asp
NS2	52	Leu	Met

### The HA gene determines the high virulence of the CK/1180 virus in mice.

To avoid any “gain of function” concerns (33), we only used the lethal virus CK/1180 as the backbone to generate the reassortants, each containing one gene derived from CK/1214, as described previously (10), and tested their replication and pathogenicity in mice. The virulence of the reassortants that contained the PB2, PB1, PA, NA, M, or NS gene of CK/1214 was attenuated 5 to 50 times (MLD_50_, 3.5 to 4.5 log_10_ EID_50_) relative to that of the rescued CK/1180 virus (MLD_50_, 2.8 log_10_ EID_50_) (Fig. 1C and Table 1). Viruses were detected in all four tested organs of the mice infected with the reassortants that contained the PB2, PB1, PA, NA, or M gene of CK/1214 and in the lungs, spleens, and kidneys (but not brains) of the mice infected with the reassortant containing the NS gene of CK/1214 (Table 1). The reassortant bearing the HA gene of CK/1214 in the CK/1180 background mainly replicated in the lungs (a low level of virus was detected in the spleens of two of three infected mice) and was dramatically attenuated (3,981-fold), with an MLD_50_ of 6.4 log_10_ EID_50_ compared with that for the R-CK/1180 virus (Table 1). These results indicate that the HA gene plays an important role in the high virulence of the CK/1180 virus in mice.

### The amino acids at positions 158 to 160 in the HA protein of CK/1180 form a glycosylation site.

**FIG 2:**
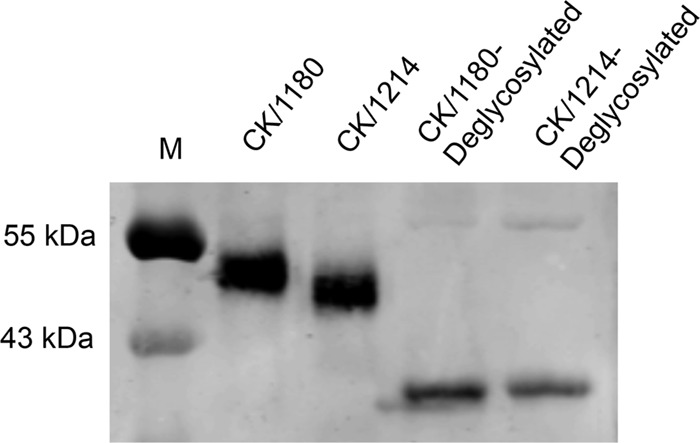
Mobility analyses of HA proteins. Lysates of H5N1 viruses treated or not with PNGase F were incubated with chicken anti-H5N1 antiserum. Protein bands were detected by using an Odyssey infrared imaging system after incubation with IRDyeTM700DX-conjugated secondary antibody. The locations of the marker proteins (M) are indicated on the left.

The predicted HA protein sequences of the CK/1180 and CK/1214 viruses differ by a single amino acid at position 158 (Table 2) (H3 numbering). Asparagine (N) at this position of the HA protein of CK/1180 virus forms a potential glycosylation site—NST—at amino acids 158 to 160, whereas there is no such site at the corresponding amino acid positions in the HA protein of the CK/1214 virus. To investigate whether N-linked glycosylation indeed occurs at this potential site, we performed Western blot analysis of HA polypeptides of CK/1180 and CK/1214 treated or not with peptide-*N*-glycosidase F (PNGase F) enzyme. We found that the HA1 polypeptide of CK/1180 exhibited decreased mobility relative to that of CK/1214, whereas when the HA1 polypeptides of the two viruses were deglycosylated with PNGase F, they showed similar mobilities (Fig. 2). These results confirm that the potential N-linked glycosylation site at amino acid positions 158 to 160 in the HA protein of the CK/1180 virus is glycosylated. Analysis of viral replication in mice showed that CK/1180 virus with this glycosylation in its HA protein systemically replicated in mice, and the loss of this glycosylation (CK/1180-1214HA virus) resulted in mainly viral replication in the lung (Table 1). These findings suggest that glycosylation at positions 158 to 160 in the HA protein promotes H5N1 virus systemic spread in mice and thereby contributes to its higher virulence in mice.

### Glycosylation at positions 158 to 160 of HA increase VLP formation and virus yield.

**FIG 3:**
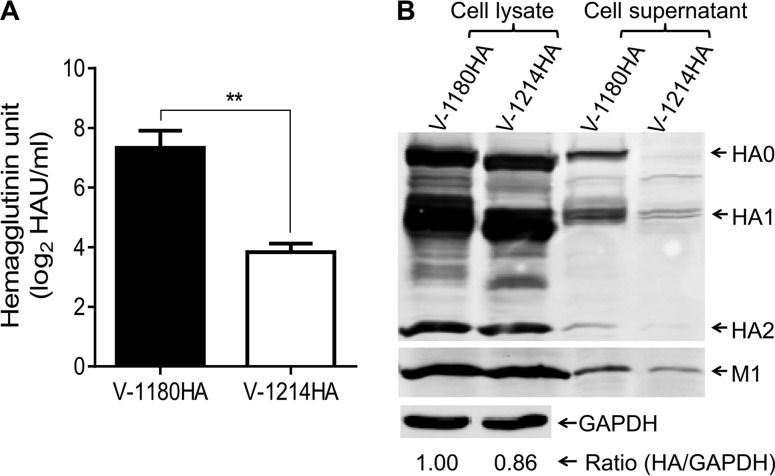
VLP formation. (A) VLPs were prepared as described in Materials and Methods. At 48 h posttransfection, the supernatants and cells were harvested. The supernatants were concentrated through a 30% sucrose cushion and then their hemagglutinin units were analyzed by using a hemagglutination assay. Data are presented as means ± SD (*n* = 3). **, *P* < 0.01 (Student's *t* test). (B) The concentrates and cell lysate were analyzed by SDS-PAGE followed by immunoblotting to detect viral proteins; the grayscale value of each band was measured by using ImageJ_1.50a software.

Virus-like particles (VLPs) of influenza viruses are extensively used to investigate viral assembly and budding efficiency (34–36). To examine the effect of the glycosylation at positions 158 to 160 of HA on VLP formation, we produced two different VLPs (designated V-1214HA and V-1180HA) and compared their respective productivities. Of note, the only difference between the two VLP particles was the glycosylation at positions 158 to 160 of the HA proteins. We found that V-1180HA produced a higher level of the hemagglutinin unit in the cell supernatants than did V-1214HA (Fig. 3A); also, more HA and M1 proteins were detected from V-1180HA in the cell supernatants (Fig. 3B) than from V-1214HA, indicating that the productivity of V-1180HA was considerably higher than that of V-1214HA.

**FIG 4:**
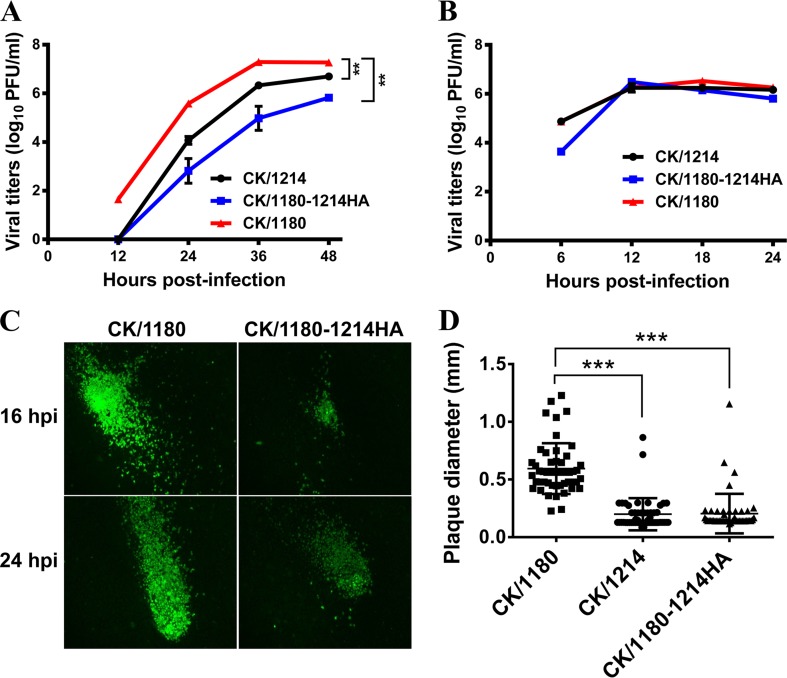
Growth kinetics of viruses in MDCK cells. Growth curves of CK/1180, CK/1214, and CK/1180-1214HA in MDCK cells infected at multiplicities of infection (MOI) of 0.001 (A) and 5 (B) are shown. Viruses were inoculated into MDCK cells. At the indicated times after infection, virus titers in the supernatant were determined by means of plaque assays. The reported values are means ± SD from two independent experiments. **, *P* < 0.01 (multiple *t* tests in GraphPad Prism 6). (C) Viruses were inoculated into MDCK cells at an MOI of 0.001. At the indicated times after infection, the cells were fixed with 4% paraformaldehyde in PBS and then analyzed with an anti-HA antibody in an immunofluorescence assay. (D) Plaque diameters of CK/1180, CK/1214, and CK/1180-1214HA viruses in MDCK cells. Plaque assays were produced under standard conditions and stained with 0.1% crystal violet. The diameters of 50 random plaques were measured for each virus. ***, *P* < 0.001 (Student's *t* test).

**FIG 5:**
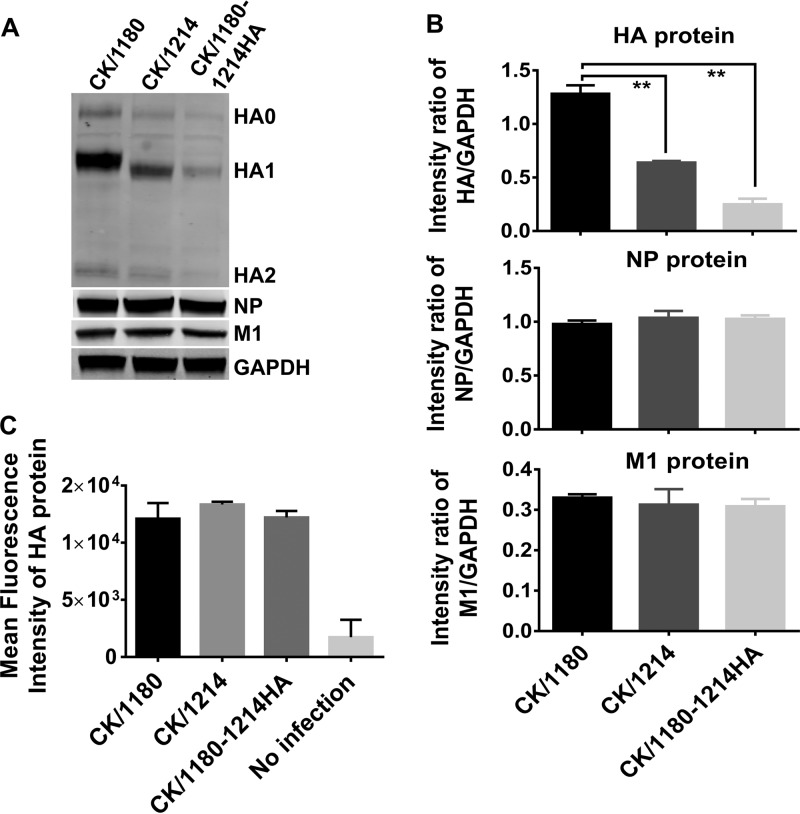
Expression of viral proteins in infected MDCK cells. MDCK cells were infected with viruses at an MOI of 5. The infected cells were collected at 12 h postinfection, lysed in SDS loading buffer, and further analyzed by Western blotting (A and B). (A) Protein bands of HA, NP, and M1 proteins detected by Western blotting. The intensity of each band was measured by using ImageJ software, and relative intensity ratios of HA, NP, and M1 compared with that of GAPDH were calculated (B). The infected cells were digested with trypsin to obtain a single cell suspension at 12 h postinfection. The samples were stained with rabbit monoclonal antibody to influenza A virus H5N1 HA protein and Alexa Fluor 488-conjugated goat anti-rabbit IgG (H+L) secondary antibody and then detected on a FACSAria II (BD Biosciences). (C) Mean fluorescence intensity of HA protein was analyzed with FlowJo X 10.0.7r2 (Tree Star, San Carlos, CA). Data are presented as means ± SD (*n* = 3). **, *P* < 0.01 (Student's *t* test).

**FIG 6:**
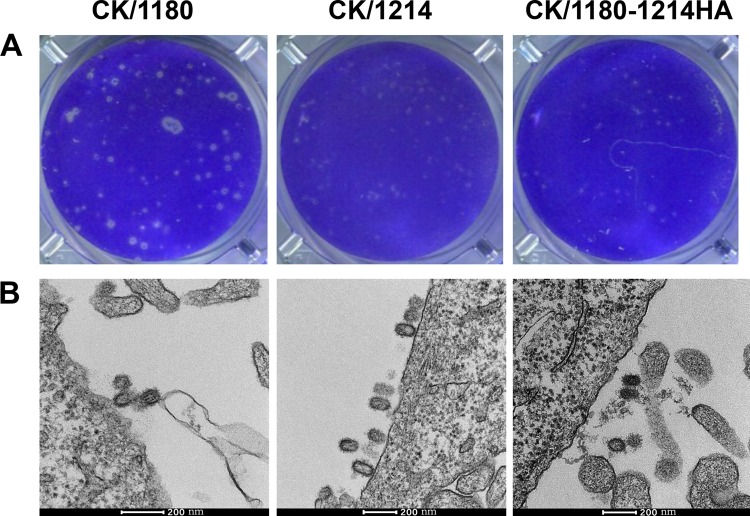
Plaque phenotypes and virus morphology in MDCK cells. (A) Plaque phenotypes of CK/1180, CK/1214, and CK/1180-1214HA viruses. Plaque assays were produced under standard conditions and stained with 0.1% crystal violet. (B) Virus morphology of CK/1180, CK/1214, and CK/1180-1214HA viruses. MDCK cells were infected with viruses at an MOI of 5. At 10 h postinfection, the cells were collected and prepared for virion morphology analysis by using transmission electron microscopy.

Next, we compared the levels of replication of CK/1180, CK/1214, and CK/1180-1214HA in MDCK cells. We found that CK/1180 replicated more efficiently than CK/1214 and CK/1180-1214HA at the desired times at a multiplicity of infection (MOI) of 0.001 (Fig. 4A), even though there was no notable difference in viral growth at an MOI of 5 (Fig. 4B). Analysis of viral proteins, including HA, NP, and M1, in the cell lysates showed that HA expression was much higher in MDCK cells infected with CK/1180 than in those infected with CK/1180-1214HA at 12 h postinfection (hpi) at an MOI of 5, but there was no difference in NP and M1 expression (Fig. 5A and B). Flow cytometric analysis of HA expression on the membrane of infected MDCK cells showed that there was no difference among CK/1180-, CK/1214-, and CK/1180-1214HA-infected MDCK cells (Fig. 5C). We checked the viral plaque phenotypes of CK/1180 and CK/1180-1214HA in MDCK cells by using an immunofluorescence assay and found that CK/1180 formed much bigger rocket-like plaques in MDCK cells than CK/1180-1214HA and displayed stronger diffusivity during replication in cells (Fig. 4C). The plaque diameters of CK/1180 were significantly larger than those of CK/1214 and CK/1180-1214HA as determined by using the standard plaque assay (37) (Fig. 4D and 6A). We then examined the MDCK cells infected with CK/1180, CK/1214, and CK/1180-1214HA at an MOI of 5 at 10 hpi by using ultrathin-section electron microscopy as described previously (38), and we found no difference in the morphology of the virions (Fig. 6B). Together, these results demonstrate that glycosylation at positions 158 to 160 of HA increases the plaque size and replication capacity of H5N1 virus in mammalian cells.

### Glycosylation at positions 158 to 160 of HA enhances the immune and inflammatory processes in infected mouse lung.

**FIG 7:**
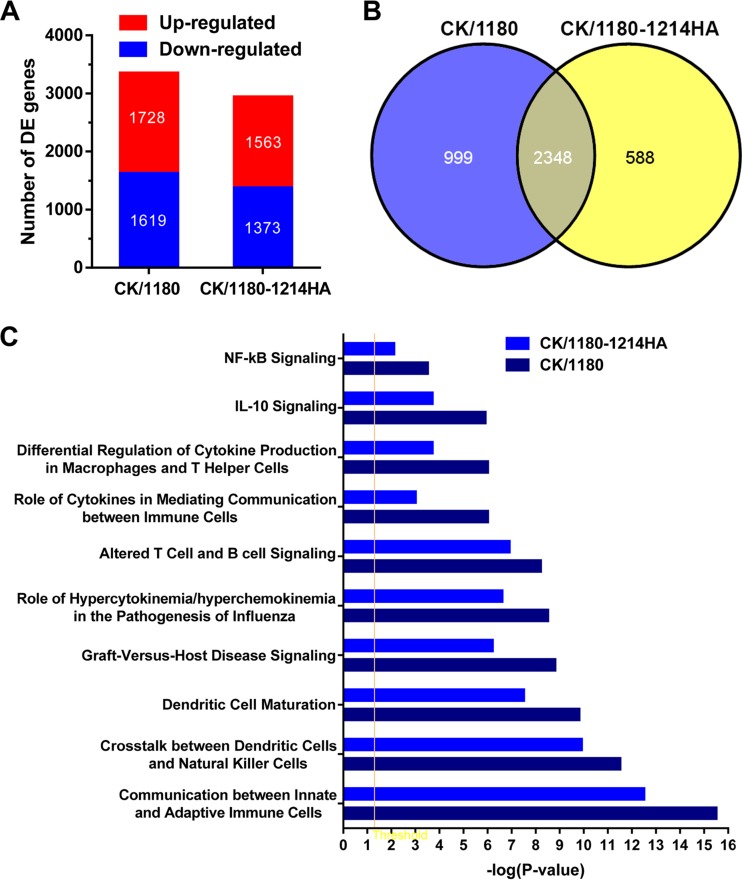
Microarray analysis of gene expression in mouse lungs. (A) Number of differentially expressed (DE) genes during infection with CK/1180 and CK/1180-1214HA relative to that in mock-infected mice (*P* < 0.05; fold change > 2). (B) Venn diagram showing the distribution of DE genes during infection with CK/1180 and CK/1180-1214HA. (C) Top canonical pathways of DE genes in mouse lungs infected with CK/1180 and CK/1180-1214HA.

**FIG 8:**
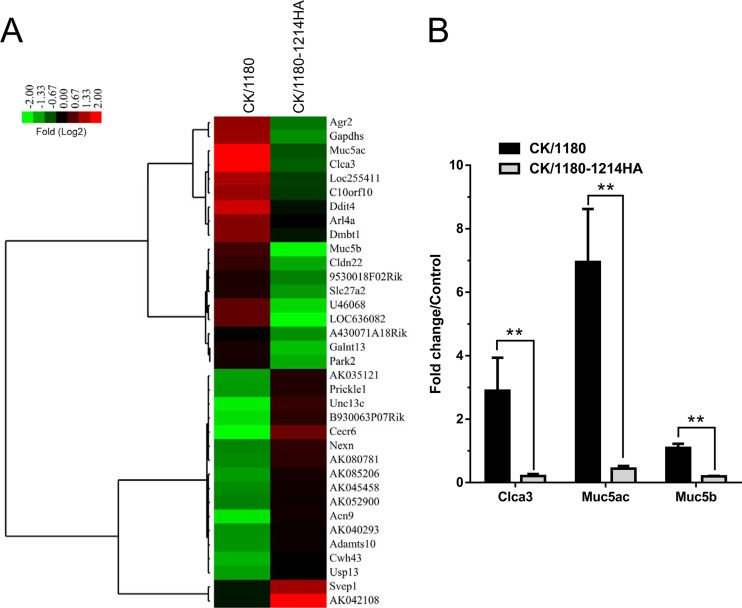
DE genes with converse regulation in mouse lungs. (A) Clustering of DE genes with converse regulation is represented on the basis of the log_2_ ratio of expression values relative to those of mock-infected mice. Red and green indicate increases and decreases, respectively. Clustering was performed with the hierarchical unweighted-pair group method using average linkages (UPGMA), with a Euclidean distance similarity measure. (B) Validation of microarray gene expression data for Clca3, Muc5ac, and Muc5b by means of qRT-PCR analysis. All values were normalized to GAPDH and are expressed as fold change compared with controls. Data are presented as means ± SD (*n* = 3). **, *P* < 0.01 (Student's *t* test).

To assess the effect of glycosylation of HA on the host response, we performed gene expression profiling in the lungs of CK/1180-, CK/1180-1214HA-, and phosphate-buffered saline (PBS)-inoculated mice by means of a microarray analysis. A total of 3,347 genes were observed to be significantly differentially expressed (*P* < 0.05; fold change > 2) during CK/1180 infection; the corresponding number was 2,936 for CK/1180-1214HA (Fig. 7A). A total of 2,348 differentially expressed (DE) genes were common to the two viruses (Fig. 7B). Canonical pathway analysis demonstrated that CK/1180 preferred to regulate higher immune response pathways than did CK/1180-1214HA, which included innate immune and adaptive immune responses (Fig. 7C). Thirty-five DE genes whose regulation was diametrically opposed between the CK/1180 and CK/1180-1214HA infections were identified (Fig. 8A); these included some mucin-related genes. For example, Clca3, a putative calcium-activated chloride channel involved in the regulation of mucus production and/or secretion (39, 40), and Muc5ac were upregulated after CK/1180 infection but downregulated after CK/1180-1214HA infection (Fig. 8A). By using quantitative PCR, we validated the expression of Clca3, Muc5ac, and Muc5b; Fig. 8B shows their fold changes in expression compared with control levels. These expression patterns are consistent with the results of the microarray analysis.

## DISCUSSION

By using two HPAI H5N1 viruses (CK/1180 and CK/1214) with similar genomes but different pathogenicities in mice (31, 32), in this study, we demonstrated that a single amino acid mutation at position 158 of the HA protein had fundamental effect on the systemic replication and pathogenicity of these H5N1 influenza viruses in mice. We further found that the substitution G158N introduced an N-linked glycosylation at positions 158 to 160 of the HA protein and that this N-linked glycosylation enhanced viral productivity in infected mammalian cells and exacerbated host immune and inflammatory responses to viral infection.

The pathogenicity of influenza viruses in mammals is complex and determined by multiple viral genes (9–16, 21, 41). The HA and NS genes of human H5N1 influenza A virus contribute to its high virulence in ferrets (42), while mutations in the PA, NP, and HA genes of pandemic (H1N1) 2009 influenza virus contribute to its adaptation to mice (43). Moreover, the PB2 and HA genes affect host range and pathogenicity in mouse-adapted influenza A virus (44). In the present study, we found that a single mutation at position 158 of HA has a major effect on the pathogenicity of H5N1 viruses in mice, with the other genes contributing only slightly to the virulence and to different extents (Table 1).

Protein glycosylation is gradually being recognized as an important means of evolution for influenza viruses (45, 46). Sun et al. stated that the number of glycosylation sites in HA protein increases during the evolution of human seasonal influenza viruses (47). The acquisition of potential glycosylation sites is an effective way for influenza viruses to escape positive selective pressures from the host (45, 48, 49). In the present study, we found that CK/1180, with an additional glycosylation site at positions 158 to 160 of HA, showed higher pathogenicity in mice, consistent with a previous report (50). Natural cytotoxicity receptors (NCRs), such as NKp46 (NCR1), NKp44 (NCR2), and NKp30 (NCR3), are important natural killer (NK) cell triggering receptors (51). NKp46 and NKp44 have been shown to functionally interact with the HA proteins of different influenza viruses during NK cell-mediated virus clearance (52, 53). We therefore speculate that the additional glycosylation site at positions 158 to 160 in the HA protein of CK/1180 may affect virus clearance mediated by NK cells by blocking the interaction of NKp46 or NKp44 with HA protein.

**FIG 9:**
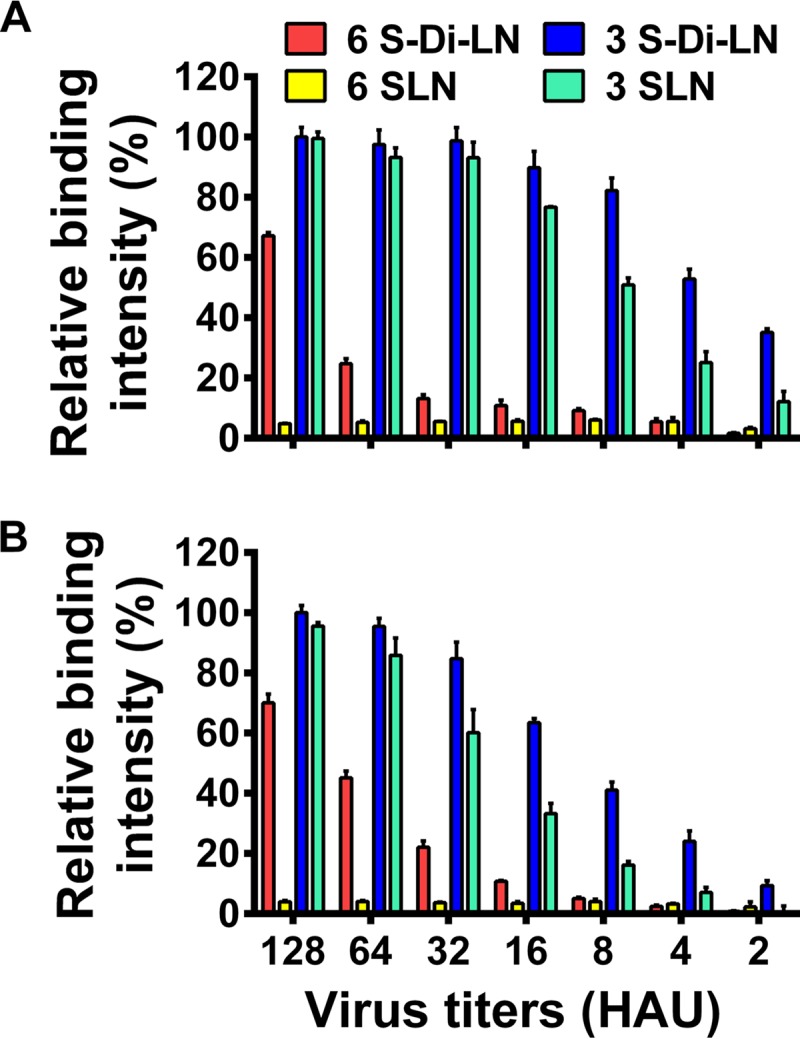
Glycan binding specificity of H5N1 viruses. The receptor specificity of influenza virus was analyzed by using a direct solid-phase assay as described previously (54). Two α-2,6 glycans, Neu5Aca2-6Galb1-4GlcNAcb-SpNH-LC-LC-biotin (6 SLN) and Neu5Aca2-6[Galb1-4GlcNAcb1-3]2b-SpNH-LC-LC-biotin (6 S-Di-LN), and two α-2,3 glycans, Neu5Aca2-3Galb1-4GlcNAcb-SpNHLC-LC-biotin (3 SLN) and Neu5Aca2-3[Galb1-4GlcNAcb1-3]2b-SpNH-LC-LC-biotin (3 S-Di-LN), were used. (A) CK/1180 virus. (B) CK/1214 virus.

The glycosylation at positions 158 to 160 in the HA protein has been documented to affect the biological properties of influenza virus in several aspects. The loss of this glycosylation site contributes to higher viral replication in the upper respiratory tract of ferrets (6), bestows upon the HA protein the ability to bind to α-2,6 glycans, and facilitates the direct contact transmission of H5N1 virus in guinea pigs (54) and the airborne transmission of influenza virus in ferrets (6, 7). In our present study, although the loss of the glycosylation at positions 158– to 160 in the HA significantly attenuated our H5N1 virus in mice, it did not alter the receptor binding preference (Fig. 9) or the viral replication in mouse nasal tissues of these viruses (Table 1, CK/1180 and CK/1180-1214HA). Moreover, we found that deletion of this glycosylation site just slightly reduced the virulence in mice of another highly pathogenic H5N1 virus A/duck/Hunan/49/05 (26) and decreased its systemic replication (Table 1). We therefore speculate that the role of glycosylation of the HA protein in viral pathogenicity to mammals and in receptor binding specificity may differ depending on the individual virus subtype or strain.

Host-specific traits and differences in host immune responses contribute to both infection and clinical outcomes. An exaggerated innate response, with early recruitment of inflammatory leukocytes to the lung, contributed to the morbidity associated with infection with the 1918 influenza virus (55). Recently, a significant association was reported between excessive early cytokine responses to avian H5N1 infection, immune cell recruitment, and poor outcome (30). In the present study, we found that CK/1180 was highly pathogenic in mice but that the loss of HA glycosylation at positions 158 to 160 significantly attenuated this pathogenicity. Gene expression profiling demonstrated that CK/1180 induced a stronger host response in the lungs of mice than that induced by CK/1180-1214HA, including immune and inflammatory responses. Mucin-related proteins modulate the rheological properties of airways and participate in lung defense (56). However, hypersecretion of airway mucus can increase the mortality associated with disease (57, 58). Influenza virus infections may induce profound disturbances of the mucociliary system (59). In the present study, CK/1180 infection induced higher expression of mucin-related proteins in mouse lungs than CK/1180-1214HA, which may explain, in part, the increased lethality of CK/1180 to mice. These findings further our understanding of the determinants of pathogenicity of H5N1 viruses in mammals.

## MATERIALS AND METHODS

### Cells and viruses.

Human embryonic kidney cells (293T) were maintained in Dulbecco's modified Eagle's medium (DMEM) supplemented with 10% fetal bovine serum plus antibiotics, and Madin-Darby canine kidney (MDCK) cells were maintained in minimal essential medium (MEM) supplemented with 5% newborn calf serum. All cells were incubated at 37°C in 5% CO_2_. Two HPAI H5N1 viruses, CK/1180 and CK/1214, were propagated in 10-day-old specific-pathogen-free (SPF) embryonated chicken eggs and stored at −80°C until use.

### Construction of plasmids.

The construction of plasmids for virus rescue was performed as described previously (12). The protein expression plasmids for HA, NA, and M1 of influenza virus were generated by inserting their cDNAs between the SacI and NheI restriction enzyme sites of the pCAGGS plasmid vector for the production of virus-like particles (VLPs). The primer sequences are available upon request. All of the constructs were completely sequenced to ensure the absence of unwanted mutations.

### Generation of reverse genetic reassortant viruses.

Reassortant viruses were generated by using an eight-plasmid reverse genetics system as described previously (12, 24). The rescued viruses were detected by using a hemagglutination assay and were fully sequenced to ensure the absence of unwanted mutations.

### Mouse experiments.

Groups of eight 6-week-old female BALB/c mice (Beijing Experimental Animal Center) were lightly anesthetized with CO_2_ and inoculated intranasally with 10^6^ 50% egg infectious doses (EID_50_) of H5N1 influenza viruses in a 50-μl volume. Three mice in each group were euthanized on day 3 postinfection. Organs, including the lungs, kidneys, spleens, and brains, were collected and titrated for virus infectivity in eggs as described previously (12). The remaining five mice were monitored daily for 14 days for weight loss and mortality. To determine the MLD_50_ of viruses that caused lethal infection of mice, six groups of five mice were inoculated intranasally with 10-fold serial dilutions containing 10^1^ to 10^6^ EID_50_ or 10^7^ EID_50_ of virus in a 50-μl volume. The MLD_50_ was calculated by using the method of Reed and Muench (60).

### Deglycosylation using PNGase F and Western blot analysis.

Deglycosylation was performed by using PNGase F enzyme (New England BioLabs). Virus was concentrated by ultracentrifugation and purified by sucrose gradient centrifugation as previously described (54). The virus concentrates were denatured and then deglycosylated with the PNGase F enzyme according to the manufacturer's instructions. Virus samples were analyzed by use of SDS-polyacrylamide gel electrophoresis (SDS-PAGE) and Western blotted with chicken antisera induced by the pCAGGS-HA DNA vaccine (61) and IRDyeTM700DX-conjugated goat anti-chicken secondary antibody (Rockland). Protein bands were detected by using an Odyssey infrared imaging system (Li-Cor Biosciences, Lincoln, NE).

### Viral replication in MDCK cells.

MDCK cells were infected with viruses at a multiplicity of infection (MOI) of 5 or 0.001. Virus-containing culture supernatants were collected at the indicated time points and titrated in MDCK cells. The infected cells were collected, lysed in SDS loading buffer, and further analyzed by Western blotting. To measure HA expression on the membrane of the infected cells, the cells were infected with viruses at an MOI of 5 and then were digested with trypsin to obtain a single cell suspension at 12 hpi. The samples were stained with a rabbit monoclonal antibody to influenza A virus H5N1 HA protein (11048-RM07; Sino Biological Inc.) and with Alexa Fluor 488-conjugated goat anti-rabbit IgG (H+L) secondary antibody (A-11034; Thermo Fisher Scientific). Data were acquired on a FACSAria II (BD Biosciences), and the mean fluorescence intensity (MFI) was analyzed with FlowJo × 10.0.7r2 (Tree Star, San Carlos, CA).

### Immunofluorescence.

MDCK cells were grown in 6-well plates and infected with virus at an MOI of 0.001. At the desired time points postinfection, the cells were fixed with PBS containing 4% paraformaldehyde. After being blocked with 5% bovine serum albumin (BSA) in PBS, the cells were incubated at room temperature for 2 h with chicken antisera induced by the pCAGGS-HA DNA vaccine. The cells were then washed three times with PBS plus Tween 20 (PBS-T) and incubated for 1 h with fluorescein isothiocyanate (FITC)-coupled goat anti-chicken secondary antibody. After that, the cells were washed three times with PBS-T and were observed with a fluorescence microscope (Carl Zeiss, Germany).

### VLP formation.

Influenza VLPs were produced in 293T cells by coexpression of influenza virus HA, NA, and M1 proteins using a plasmid-based VLP system as described previously (62). Briefly, 293T cells were transfected with pCAGGS plasmids expressing the viral proteins HA, NA, and M1 by using Lipofectamine and Plus reagents (Invitrogen) according to the manufacturer's instructions. As described previously (34), the released VLPs in the cell supernatant were harvested by using ultracentrifugation at 48 h posttransfection. The pellet (after ultracentrifugation) and cells were lysed in SDS sample buffer solution (Wako) with 100 mM dithiothreitol and subjected to Western blotting.

### Microarray analysis.

For all gene expression analyses, groups of three mice were inoculated with PBS or 10^6^ EID_50_ of CK/1180 or CK/1180-1214HA. Total RNA was extracted from lung tissues on day 3 postinfection by using a Qiagen RNeasy kit. The microarray assay was performed by using a low-RNA-input linear amplification kit (Agilent Technologies, Santa Clara, CA) and Agilent's whole mouse genome microarray kit, 4 × 44 K (G4122F), as described elsewhere (25). Student's *t* test and significant analysis of microarray (SAM) were performed to determine the genes that had significantly different expression levels (*P* < 0.05 and >2-fold change) upon infection compared with levels in the PBS-inoculated group. Ingenuity Pathways Analysis (IPA) (Ingenuity Systems, Redwood City, CA) was applied for functional and network analyses of significantly differentially expressed genes.

### Validation of array expression data by qRT-PCR.

Quantitative real-time PCR (qRT-PCR) was performed to validate the changes in expression of selected genes detected by microarray as described previously (32). Glyceraldehyde-3-phosphate dehydrogenase (GAPDH) was used for normalization. Primer sequences and protocols for the qRT-PCR are available upon request. Tests were performed in duplicate in at least three separate experiments, and relative RNA levels were determined by using the control group as the reference sample.

### Laboratory facility.

Studies with HPAI H5N1 viruses were conducted in a biosafety level 3 laboratory approved for such use by the Committee on the Ethics of Animal Experiments of the Harbin Veterinary Research Institute, Chinese Academy of Agricultural Sciences (approval number BRDW-XBS-09). All studies were carried out in strict accordance with the recommendations in the *Guide for the Care and Use of Laboratory Animals* (63) of the Ministry of Science and Technology of the People's Republic of China.

### Accession number(s).

All primary expression microarray data have been deposited in the Gene Expression Omnibus database (http://www.ncbi.nlm.nih.gov/projects/geo/) under the accession number GSE89246.

## References

[1] Swayne DE 2012 Impact of vaccines and vaccination on global control of avian influenza. Avian Dis 56:818–828. doi:10.1637/10183-041012-Review.1. 23402099

[2] Li C, Bu Z, Chen H 2014 Avian influenza vaccines against H5N1 ‘bird flu.’ Trends Biotechnol 32:147–156. doi:10.1016/j.tibtech.2014.01.001. 24491922

[3] Kandun IN, Wibisono H, Sedyaningsih ER, Yusharmen Hadisoedarsuno W, Purba W, Santoso H, Septiawati C, Tresnaningsih E, Heriyanto B, Yuwono D, Harun S, Soeroso S, Giriputra S, Blair PJ, Jeremijenko A, Kosasih H, Putnam SD, Samaan G, Silitonga M, Chan KH, Poon LL, Lim W, Klimov A, Lindstrom S, Guan Y, Donis R, Katz J, Cox N, Peiris M, Uyeki TM 2006 Three Indonesian clusters of H5N1 virus infection in 2005. N Engl J Med 355:2186–2194. doi:10.1056/NEJMoa060930. 17124016

[4] Olsen SJ, Ungchusak K, Sovann L, Uyeki TM, Dowell SF, Cox NJ, Aldis W, Chunsuttiwat S 2005 Family clustering of avian influenza A (H5N1). Emerg Infect Dis 11:1799–1801. doi:10.3201/eid1111.050646. 16422010 PMC3367331

[5] World Health Organization. 2006 Human cases of influenza A(H5N1) infection in eastern Turkey, December 2005–January 2006. Wkly Epidemiol Rec 81:410–416. 17072997

[6] Imai M, Watanabe T, Hatta M, Das SC, Ozawa M, Shinya K, Zhong G, Hanson A, Katsura H, Watanabe S, Li C, Kawakami E, Yamada S, Kiso M, Suzuki Y, Maher EA, Neumann G, Kawaoka Y 2012 Experimental adaptation of an influenza H5 HA confers respiratory droplet transmission to a reassortant H5 HA/H1N1 virus in ferrets. Nature 486:420–428. 22722205 10.1038/nature10831 PMC3388103

[7] Herfst S, Schrauwen EJ, Linster M, Chutinimitkul S, de Wit E, Munster VJ, Sorrell EM, Bestebroer TM, Burke DF, Smith DJ, Rimmelzwaan GF, Osterhaus AD, Fouchier RA 2012 Airborne transmission of influenza A/H5N1 virus between ferrets. Science 336:1534–1541. doi:10.1126/science.1213362. 22723413 PMC4810786

[8] Zhang Y, Zhang Q, Kong H, Jiang Y, Gao Y, Deng G, Shi J, Tian G, Liu L, Liu J, Guan Y, Bu Z, Chen H 2013 H5N1 hybrid viruses bearing 2009/H1N1 virus genes transmit in guinea pigs by respiratory droplet. Science 340:1459–1463. doi:10.1126/science.1229455. 23641061

[9] Fan S, Deng G, Song J, Tian G, Suo Y, Jiang Y, Guan Y, Bu Z, Kawaoka Y, Chen H 2009 Two amino acid residues in the matrix protein M1 contribute to the virulence difference of H5N1 avian influenza viruses in mice. Virology 384:28–32. doi:10.1016/j.virol.2008.11.044. 19117585

[10] Hatta M, Gao P, Halfmann P, Kawaoka Y 2001 Molecular basis for high virulence of Hong Kong H5N1 influenza A viruses. Science 293:1840–1842. doi:10.1126/science.1062882. 11546875

[11] Jiao P, Tian G, Li Y, Deng G, Jiang Y, Liu C, Liu W, Bu Z, Kawaoka Y, Chen H 2008 A single-amino-acid substitution in the NS1 protein changes the pathogenicity of H5N1 avian influenza viruses in mice. J Virol 82:1146–1154. doi:10.1128/JVI.01698-07. 18032512 PMC2224464

[12] Li Z, Chen H, Jiao P, Deng G, Tian G, Li Y, Hoffmann E, Webster RG, Matsuoka Y, Yu K 2005 Molecular basis of replication of duck H5N1 influenza viruses in a mammalian mouse model. J Virol 79:12058–12064. doi:10.1128/JVI.79.18.12058-12064.2005. 16140781 PMC1212590

[13] Li Z, Jiang Y, Jiao P, Wang A, Zhao F, Tian G, Wang X, Yu K, Bu Z, Chen H 2006 The NS1 gene contributes to the virulence of H5N1 avian influenza viruses. J Virol 80:11115–11123. doi:10.1128/JVI.00993-06. 16971424 PMC1642184

[14] Seo SH, Hoffmann E, Webster RG 2004 The NS1 gene of H5N1 influenza viruses circumvents the host anti-viral cytokine responses. Virus Res 103:107–113. doi:10.1016/j.virusres.2004.02.022. 15163498

[15] Song MS, Pascua PN, Lee JH, Baek YH, Lee OJ, Kim CJ, Kim H, Webby RJ, Webster RG, Choi YK 2009 The polymerase acidic protein gene of influenza A virus contributes to pathogenicity in a mouse model. J Virol 83:12325–12335. doi:10.1128/JVI.01373-09. 19793828 PMC2786751

[16] Zhu Q, Yang H, Chen W, Cao W, Zhong G, Jiao P, Deng G, Yu K, Yang C, Bu Z, Kawaoka Y, Chen H 2008 A naturally occurring deletion in its NS gene contributes to the attenuation of an H5N1 swine influenza virus in chickens. J Virol 82:220–228. doi:10.1128/JVI.00978-07. 17942562 PMC2224367

[17] Pappas C, Aguilar PV, Basler CF, Solorzano A, Zeng H, Perrone LA, Palese P, Garcia-Sastre A, Katz JM, Tumpey TM 2008 Single gene reassortants identify a critical role for PB1, HA, and NA in the high virulence of the 1918 pandemic influenza virus. Proc Natl Acad Sci U S A 105:3064–3069. doi:10.1073/pnas.0711815105. 18287069 PMC2268585

[18] Kobasa D, Takada A, Shinya K, Hatta M, Halfmann P, Theriault S, Suzuki H, Nishimura H, Mitamura K, Sugaya N, Usui T, Murata T, Maeda Y, Watanabe S, Suresh M, Suzuki T, Suzuki Y, Feldmann H, Kawaoka Y 2004 Enhanced virulence of influenza A viruses with the haemagglutinin of the 1918 pandemic virus. Nature 431:703–707. doi:10.1038/nature02951. 15470432

[19] Kawaoka Y, Webster RG 1988 Sequence requirements for cleavage activation of influenza virus hemagglutinin expressed in mammalian cells. Proc Natl Acad Sci U S A 85:324–328. doi:10.1073/pnas.85.2.324. 2829180 PMC279540

[20] Suguitan AL Jr, Matsuoka Y, Lau YF, Santos CP, Vogel L, Cheng LI, Orandle M, Subbarao K 2012 The multibasic cleavage site of the hemagglutinin of highly pathogenic A/Vietnam/1203/2004 (H5N1) avian influenza virus acts as a virulence factor in a host-specific manner in mammals. J Virol 86:2706–2714. doi:10.1128/JVI.05546-11. 22205751 PMC3302284

[21] Horimoto T, Rivera E, Pearson J, Senne D, Krauss S, Kawaoka Y, Webster RG 1995 Origin and molecular changes associated with emergence of a highly pathogenic H5N2 influenza virus in Mexico. Virology 213:223–230. doi:10.1006/viro.1995.1562. 7483266

[22] Mehle A, Doudna JA 2009 Adaptive strategies of the influenza virus polymerase for replication in humans. Proc Natl Acad Sci U S A 106:21312–21316. doi:10.1073/pnas.0911915106. 19995968 PMC2789757

[23] Yamada S, Hatta M, Staker BL, Watanabe S, Imai M, Shinya K, Sakai-Tagawa Y, Ito M, Ozawa M, Watanabe T, Sakabe S, Li C, Kim JH, Myler PJ, Phan I, Raymond A, Smith E, Stacy R, Nidom CA, Lank SM, Wiseman RW, Bimber BN, O'Connor DH, Neumann G, Stewart LJ, Kawaoka Y 2010 Biological and structural characterization of a host-adapting amino acid in influenza virus. PLoS Pathog 6:e1001034. doi:10.1371/journal.ppat.1001034. 20700447 PMC2916879

[24] Song J, Feng H, Xu J, Zhao D, Shi J, Li Y, Deng G, Jiang Y, Li X, Zhu P, Guan Y, Bu Z, Kawaoka Y, Chen H 2011 The PA protein directly contributes to the virulence of H5N1 avian influenza viruses in domestic ducks. J Virol 85:2180–2188. doi:10.1128/JVI.01975-10. 21177821 PMC3067757

[25] Hu J, Hu Z, Song Q, Gu M, Liu X, Wang X, Hu S, Chen C, Liu H, Liu W, Chen S, Peng D, Liu X 2013 The PA-gene-mediated lethal dissemination and excessive innate immune response contribute to the high virulence of H5N1 avian influenza virus in mice. J Virol 87:2660–2672. doi:10.1128/JVI.02891-12. 23255810 PMC3571398

[26] Song J, Xu J, Shi J, Li Y, Chen H 2015 Synergistic effect of S224P and N383D substitutions in the PA of H5N1 avian influenza virus contributes to mammalian adaptation. Sci Rep 5:10510. doi:10.1038/srep10510. 26000865 PMC4441148

[27] Baskin CR, Bielefeldt-Ohmann H, Tumpey TM, Sabourin PJ, Long JP, Garcia-Sastre A, Tolnay AE, Albrecht R, Pyles JA, Olson PH, Aicher LD, Rosenzweig ER, Murali-Krishna K, Clark EA, Kotur MS, Fornek JL, Proll S, Palermo RE, Sabourin CL, Katze MG 2009 Early and sustained innate immune response defines pathology and death in nonhuman primates infected by highly pathogenic influenza virus. Proc Natl Acad Sci U S A 106:3455–3460. doi:10.1073/pnas.0813234106. 19218453 PMC2642661

[28] Cameron CM, Cameron MJ, Bermejo-Martin JF, Ran L, Xu L, Turner PV, Ran R, Danesh A, Fang Y, Chan PK, Mytle N, Sullivan TJ, Collins TL, Johnson MG, Medina JC, Rowe T, Kelvin DJ 2008 Gene expression analysis of host innate immune responses during lethal H5N1 infection in ferrets. J Virol 82:11308–11317. doi:10.1128/JVI.00691-08. 18684821 PMC2573250

[29] Szretter KJ, Gangappa S, Lu X, Smith C, Shieh WJ, Zaki SR, Sambhara S, Tumpey TM, Katz JM 2007 Role of host cytokine responses in the pathogenesis of avian H5N1 influenza viruses in mice. J Virol 81:2736–2744. doi:10.1128/JVI.02336-06. 17182684 PMC1866007

[30] de Jong MD, Simmons CP, Thanh TT, Hien VM, Smith GJ, Chau TN, Hoang DM, Chau NV, Khanh TH, Dong VC, Qui PT, Cam BV, Ha do Q, Guan Y, Peiris JS, Chinh NT, Hien TT, Farrar J 2006 Fatal outcome of human influenza A (H5N1) is associated with high viral load and hypercytokinemia. Nat Med 12:1203–1207. doi:10.1038/nm1477. 16964257 PMC4333202

[31] Zhao D, Liang L, Li Y, Jiang Y, Liu L, Chen H 2012 Phylogenetic and pathogenic analyses of avian influenza A H5N1 viruses isolated from poultry in Vietnam. PLoS One 7:e50959. doi:10.1371/journal.pone.0050959. 23226433 PMC3511418

[32] Zhao D, Liang L, Li Y, Liu L, Guan Y, Jiang Y, Chen H 2012 Proteomic analysis of the lungs of mice infected with different pathotypes of H5N1 avian influenza viruses. Proteomics 12:1970–1982. doi:10.1002/pmic.201100619. 22623221

[33] Duprex WP, Fouchier RA, Imperiale MJ, Lipsitch M, Relman DA 2015 Gain-of-function experiments: time for a real debate. Nat Rev Microbiol 13:58–64. 25482289 10.1038/nrmicro3405 PMC7097416

[34] Watanabe T, Kawakami E, Shoemaker JE, Lopes TJ, Matsuoka Y, Tomita Y, Kozuka-Hata H, Gorai T, Kuwahara T, Takeda E, Nagata A, Takano R, Kiso M, Yamashita M, Sakai-Tagawa Y, Katsura H, Nonaka N, Fujii H, Fujii K, Sugita Y, Noda T, Goto H, Fukuyama S, Watanabe S, Neumann G, Oyama M, Kitano H, Kawaoka Y 2014 Influenza virus-host interactome screen as a platform for antiviral drug development. Cell Host Microbe 16:795–805. doi:10.1016/j.chom.2014.11.002. 25464832 PMC4451456

[35] Gorai T, Goto H, Noda T, Watanabe T, Kozuka-Hata H, Oyama M, Takano R, Neumann G, Watanabe S, Kawaoka Y 2012 F1Fo-ATPase, F-type proton-translocating ATPase, at the plasma membrane is critical for efficient influenza virus budding. Proc Natl Acad Sci U S A 109:4615–4620. doi:10.1073/pnas.1114728109. 22393008 PMC3311379

[36] Das SC, Watanabe S, Hatta M, Noda T, Neumann G, Ozawa M, Kawaoka Y 2012 The highly conserved arginine residues at positions 76 through 78 of influenza A virus matrix protein M1 play an important role in viral replication by affecting the intracellular localization of M1. J Virol 86:1522–1530. doi:10.1128/JVI.06230-11. 22090133 PMC3264381

[37] Eisfeld AJ, Neumann G, Kawaoka Y 2014 Influenza A virus isolation, culture and identification. Nat Protoc 9:2663–2681. doi:10.1038/nprot.2014.180. 25321410 PMC5619698

[38] Noda T, Sagara H, Suzuki E, Takada A, Kida H, Kawaoka Y 2002 Ebola virus VP40 drives the formation of virus-like filamentous particles along with GP. J Virol 76:4855–4865. doi:10.1128/JVI.76.10.4855-4865.2002. 11967302 PMC136157

[39] Sabo-Attwood T, Ramos-Nino M, Bond J, Butnor KJ, Heintz N, Gruber AD, Steele C, Taatjes DJ, Vacek P, Mossman BT 2005 Gene expression profiles reveal increased mClca3 (Gob5) expression and mucin production in a murine model of asbestos-induced fibrogenesis. Am J Pathol 167:1243–1256. doi:10.1016/S0002-9440(10)61212-6. 16251409 PMC1603789

[40] Leverkoehne I, Gruber AD 2002 The murine mCLCA3 (alias gob-5) protein is located in the mucin granule membranes of intestinal, respiratory, and uterine goblet cells. J Histochem Cytochem 50:829–838. doi:10.1177/002215540205000609. 12019299

[41] McAuley JL, Hornung F, Boyd KL, Smith AM, McKeon R, Bennink J, Yewdell JW, McCullers JA 2007 Expression of the 1918 influenza A virus PB1-F2 enhances the pathogenesis of viral and secondary bacterial pneumonia. Cell Host Microbe 2:240–249. doi:10.1016/j.chom.2007.09.001. 18005742 PMC2083255

[42] Imai H, Shinya K, Takano R, Kiso M, Muramoto Y, Sakabe S, Murakami S, Ito M, Yamada S, Le MT, Nidom CA, Sakai-Tagawa Y, Takahashi K, Omori Y, Noda T, Shimojima M, Kakugawa S, Goto H, Iwatsuki-Horimoto K, Horimoto T, Kawaoka Y 2010 The HA and NS genes of human H5N1 influenza A virus contribute to high virulence in ferrets. PLoS Pathog 6:e1001106. doi:10.1371/journal.ppat.1001106. 20862325 PMC2940759

[43] Sakabe S, Ozawa M, Takano R, Iwastuki-Horimoto K, Kawaoka Y 2011 Mutations in PA, NP, and HA of a pandemic (H1N1) 2009 influenza virus contribute to its adaptation to mice. Virus Res 158:124–129. doi:10.1016/j.virusres.2011.03.022. 21458512 PMC3103651

[44] Ping J, Dankar SK, Forbes NE, Keleta L, Zhou Y, Tyler S, Brown EG 2010 PB2 and hemagglutinin mutations are major determinants of host range and virulence in mouse-adapted influenza A virus. J Virol 84:10606–10618. doi:10.1128/JVI.01187-10. 20702632 PMC2950562

[45] Vigerust DJ, Shepherd VL 2007 Virus glycosylation: role in virulence and immune interactions. Trends Microbiol 15:211–218. doi:10.1016/j.tim.2007.03.003. 17398101 PMC7127133

[46] Wei CJ, Boyington JC, Dai K, Houser KV, Pearce MB, Kong WP, Yang ZY, Tumpey TM, Nabel GJ 2010 Cross-neutralization of 1918 and 2009 influenza viruses: role of glycans in viral evolution and vaccine design. Sci Transl Med 2:24ra21. 10.1126/scitranslmed.3000799 PMC3182573 20375007

[47] Sun S, Wang Q, Zhao F, Chen W, Li Z 2011 Glycosylation site alteration in the evolution of influenza A (H1N1) viruses. PLoS One 6:e22844. doi:10.1371/journal.pone.0022844. 21829533 PMC3145772

[48] Sun S, Wang Q, Zhao F, Chen W, Li Z 2012 Prediction of biological functions on glycosylation site migrations in human influenza H1N1 viruses. PLoS One 7:e32119. doi:10.1371/journal.pone.0032119. 22355413 PMC3280219

[49] Schulze IT 1997 Effects of glycosylation on the properties and functions of influenza virus hemagglutinin. J Infect Dis 176(Suppl 1):S24–S28. 9240690 10.1086/514170

[50] Yen HL, Aldridge JR, Boon AC, Ilyushina NA, Salomon R, Hulse-Post DJ, Marjuki H, Franks J, Boltz DA, Bush D, Lipatov AS, Webby RJ, Rehg JE, Webster RG 2009 Changes in H5N1 influenza virus hemagglutinin receptor binding domain affect systemic spread. Proc Natl Acad Sci U S A 106:286–291. doi:10.1073/pnas.0811052106. 19116267 PMC2629220

[51] Moretta A, Bottino C, Vitale M, Pende D, Cantoni C, Mingari MC, Biassoni R, Moretta L 2001 Activating receptors and coreceptors involved in human natural killer cell-mediated cytolysis. Annu Rev Immunol 19:197–223. doi:10.1146/annurev.immunol.19.1.197. 11244035

[52] Mandelboim O, Lieberman N, Lev M, Paul L, Arnon TI, Bushkin Y, Davis DM, Strominger JL, Yewdell JW, Porgador A 2001 Recognition of haemagglutinins on virus-infected cells by NKp46 activates lysis by human NK cells. Nature 409:1055–1060. doi:10.1038/35059110. 11234016

[53] Ho JW, Hershkovitz O, Peiris M, Zilka A, Bar-Ilan A, Nal B, Chu K, Kudelko M, Kam YW, Achdout H, Mandelboim M, Altmeyer R, Mandelboim O, Bruzzone R, Porgador A 2008 H5-type influenza virus hemagglutinin is functionally recognized by the natural killer-activating receptor NKp44. J Virol 82:2028–2032. doi:10.1128/JVI.02065-07. 18077718 PMC2258730

[54] Gao Y, Zhang Y, Shinya K, Deng G, Jiang Y, Li Z, Guan Y, Tian G, Li Y, Shi J, Liu L, Zeng X, Bu Z, Xia X, Kawaoka Y, Chen H 2009 Identification of amino acids in HA and PB2 critical for the transmission of H5N1 avian influenza viruses in a mammalian host. PLoS Pathog 5:e1000709. doi:10.1371/journal.ppat.1000709. 20041223 PMC2791199

[55] Kobasa D, Jones SM, Shinya K, Kash JC, Copps J, Ebihara H, Hatta Y, Kim JH, Halfmann P, Hatta M, Feldmann F, Alimonti JB, Fernando L, Li Y, Katze MG, Feldmann H, Kawaoka Y 2007 Aberrant innate immune response in lethal infection of macaques with the 1918 influenza virus. Nature 445:319–323. doi:10.1038/nature05495. 17230189

[56] Barbier D, Garcia-Verdugo I, Pothlichet J, Khazen R, Descamps D, Rousseau K, Thornton D, Si-Tahar M, Touqui L, Chignard M, Sallenave JM 2012 Influenza A induces the major secreted airway mucin MUC5AC in a protease-EGFR-extracellular regulated kinase-Sp1-dependent pathway. Am J Respir Cell Mol Biol 47:149–157. doi:10.1165/rcmb.2011-0405OC. 22383584

[57] Evans CM, Kim K, Tuvim MJ, Dickey BF 2009 Mucus hypersecretion in asthma: causes and effects. Curr Opin Pulm Med 15:4–11. doi:10.1097/MCP.0b013e32831da8d3. 19077699 PMC2709596

[58] Morcillo EJ, Cortijo J 2006 Mucus and MUC in asthma. Curr Opin Pulm Med 12:1–6. doi:10.1097/01.mcp.0000198064.27586.37. 16357571

[59] Konietzko N 1986 Mucus transport and inflammation. Eur J Respir Dis Suppl 147:72–79. 3533595

[60] Reed LJ, Muench H 1938 A simple method of estimating fifty percent endpoints. Am J Hyg 27:493–497.

[61] Jiang Y, Yu K, Zhang H, Zhang P, Li C, Tian G, Li Y, Wang X, Ge J, Bu Z, Chen H 2007 Enhanced protective efficacy of H5 subtype avian influenza DNA vaccine with codon optimized HA gene in a pCAGGS plasmid vector. Antiviral Res 75:234–241. doi:10.1016/j.antiviral.2007.03.009. 17451817

[62] Chen BJ, Leser GP, Morita E, Lamb RA 2007 Influenza virus hemagglutinin and neuraminidase, but not the matrix protein, are required for assembly and budding of plasmid-derived virus-like particles. J Virol 81:7111–7123. doi:10.1128/JVI.00361-07. 17475660 PMC1933269

[63] Ministry of Science and Technology of the People's Republic of China. Guide for the care and use of laboratory animals. Ministry of Science and Technology of the People's Republic of China, Beijing, People's Republic of China. (In Chinese.)

